# Am-aza-ing antidiabetic: Mulberry dehydrogenase MnGUTB1 contributes to the biosynthesis of 1-deoxynojirimycin

**DOI:** 10.1093/plphys/kiad140

**Published:** 2023-03-06

**Authors:** Henryk Straube

**Affiliations:** Plant Physiology, American Society of Plant Biologists, USA; Faculty of Science, Department of Plant and Environmental Sciences, Section for Plant Biochemistry, University of Copenhagen, Copenhagen, Denmark

Diabetes has become a major global health emergency, and the number of people with diabetes is expected to increase to 700 million by 2045 (International Diabetes Federation 2021). Most of the currently available antidiabetic drugs have adverse effects; thus, developing new drugs is necessary and urgent. New antidiabetic drugs can be derived from natural products, such as chemicals synthesized by organisms like bacteria, fungi, and plants. Plants produce many natural products with potential use for medicinal purposes. A promising class of compounds for antihyperglycemia are azasugars, also called iminosugars, which are strong *α*-glucosidase inhibitors with the potential to slow down the digestion of carbohydrates (Watson et al. 2001).

1-Deoxynojirimycin (DNJ) is an azasugar that is already used as an antihyperglycemia drug (Ramappa et al. 2020). It is produced by several plants including mulberry (*Morus* spp.), hyacinth (*Hyacinthus orientalis*), dayflower (*Commelina communis*), and Japanese lady bell (*Adenophora triphylla*). Natural resources are currently insufficient to meet the commercial demand for DNJ (Zhang et al. 2019), and the extraction of natural products from plant material or its chemical synthesis often results in low and inconsistent yields (Pedreño and Almagro 2020). Previous studies suggest that DNJ in plants is derived from either lysine or glucose, but how exactly plants synthesize DNJ remains unknown (Shibano et al. 2004; Liu et al. 2020). Some bacteria of the genus *Bacillus* and *Streptomyces* are also capable of synthesizing DNJ from glucose via 2-amino-2-deoxy-D-mannitol (ADM) and nojirimycine (NJ) in several reactions (Clark et al. 2011).

**Figure 1.**
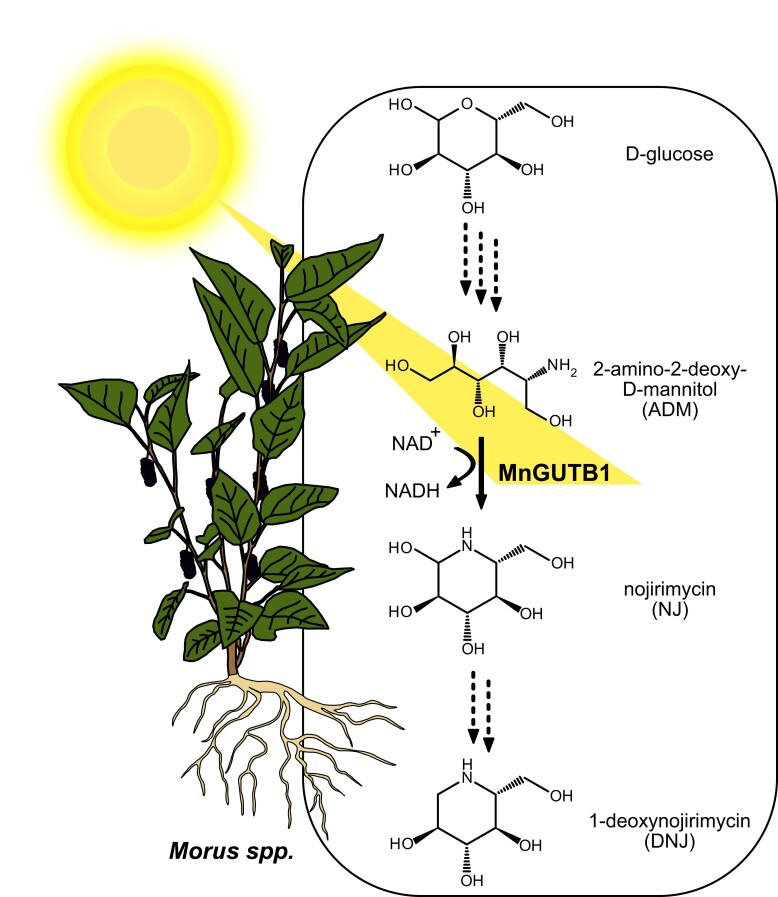
A schematic overview of the proposed 1-deoxynojirimycin (DNJ) biosynthesis pathway in mulberry (*Morus* spp.). In plants like mulberry, DNJ is likely derived from D-glucose via ADM (2-amino-2-deoxy-D-mannitol) and NJ (nojirimycin). The amino-polyol dehydrogenase MnGUTB1, identified and characterized by Yang et al. (2023), catalyzes the conversion of ADM to NJ in mulberry, an essential step in the biosynthesis of the antihyperglycemia DNJ. Dotted arrows depict reactions for which no enzymes are currently described to catalyze them in plants. The expression of MnGUTB1 is also demonstrated to be dependent on the photoperiod. A longer day leads to a more abundant MnGUTB1 transcript.

In this issue of *Plant Physiology*, Yang et al. (2023) provide clear evidence that a 2-amino-2-deoxy-D-mannitol (ADM) dehydrogenase, MnGUTB1, participates in the biosynthesis of the azasugar DNJ, catalyzing the oxidation of ADM to NJ in mulberry (Fig. 1).

An outstanding question on DNJ production in mulberry was the tissue-specific concentration of DNJ and the occurrence of potential precursors. Analyzing the amount of DNJ in 10 different tissues of mulberry, the authors identified that DNJ was most abundant in buds and younger leaves, whereas the amount of DNJ decreased in more mature leaves. They also detected ADM, a known intermediate of DNJ biosynthesis in *Bacilli*, demonstrating its existence in mulberry. As ADM is the substrate of the bacterial enzyme GutB, the authors searched the mulberry genome and identified 13 GutB homologs.

Among the 13 homologs, only MnGUTB1 contained the aspartic acid residue that is required for bacterial GutBs to properly recognize the substrate ADM. In vitro biochemical assays also demonstrated that only MnGUTB1 was able to catalyze the formation of NJ from ADM and that a mutation of the Asp residue to Asn abolished the ability to produce NJ from ADM.

Many genes involved in the biosynthesis of specialized metabolites are co-regulated at the transcript level (Jacobowitz and Weng 2020). A correlation analysis of the transcript abundance of *MnGUTB1* and the content of DNJ and its derivatives resulted in a strong positive correlation, further indicating involvement of MnGUTB1 in DNJ biosynthesis. Like DNJ, the *MnGUTB1* transcript was most abundant in buds and stems.

DNJ is not detectable in mulberry cells grown in callus culture. To identify the underlying reasons, the authors analyzed the content of NJ and ADM in mulberry calli. They found that NJ was less abundant in calli than in other tissues of mulberry while ADM concentrations were increased. Moreover, *MnGUTB1* transcription was repressed in the callus. Bioinformatic analysis showed that the promotor region of *MnGUTB1* contains several elements associated with light responsiveness. Indeed, the expression of *MnGUTB1* depended on the photoperiod (Fig. 1). A longer day resulted in increased expression of *MnGUTB1*, consistent with previous reports that DNJ content is elevated in mulberries cultivated under a longer photoperiod. Recently, two piperidine-synthase genes in *Morus alba*, Δ1-piperideine reductase 1 (*MaSDR1*) and Δ1-piperideine reductase 2 (*MaSDR2*), have been reported to be involved in the DNJ biosynthesis pathway starting from lysine (Liu et al. 2020). Interestingly, the transcripts of their homologs in *M. notabilis*, *MnSDR1* and *MnSDR2*, were more abundant in plants grown under a short photoperiod, and the expression levels of *MnSDR1* and *MnSDR2* did not correlate strongly with concentrations of DNJ or its derivatives.

Although the authors showed that MnGUTB1 likely participates in DNJ biosynthesis, in vivo evidence was still lacking. Therefore, the authors used a hairy root system to overexpress *MnGUTB1* and analyzed the transcript and metabolite content in the transgenic roots. As expected, a higher amount of the transcript led to increased DNJ concentrations. These findings were complemented by the fact that virus-induced gene silencing of *MnGUTB1* resulted in reduced *MnGUTB1* transcript amounts in mulberry leaves, leading to reduced DNJ content.

In summary, Yang et al. (2023) identified an amino-polyol dehydrogenase in mulberry that participates in the biosynthesis of antihyperglycemia azasugar DNJ. They provided evidence for its biochemical activity, presented data on tissue localization of DNJ biosynthesis, and supplied an explanation for the photoperiodical dependency of DNJ contents in mulberry. Furthermore, they confirmed their results using two in vivo approaches, indicating a connection between *MnGUTB1* transcript and DNJ abundance. The results support that the DNJ biosynthesis pathway in plants starts with C1-immination of glucose (Fig. 1), followed by oxidation and reduction to NJ. NJ can then be dehydrated and reduced to DNJ. The findings were consistent with previous results in which labeled glucose was incorporated into DNJ in *Commelina communis* (Shibano et al. 2004). Whether plants synthesize DNJ from glucose, derive DNJ from lysine via piperidine, or use both pathways remains unknown. Several enzymes involved in DNJ biosynthesis await their identification and characterization, regardless of the exact pathway. Besides the open questions, Yang et al. (2023) provide valuable insights to increase the production of DNJ by optimizing mulberry cultivation, molecular-assisted breeding, synthetic biology, and metabolic engineering.
